# Supplementary figures and images for: Expression patterns of cysteine peptidase genes across the Tribolium castaneum life cycle provide clues to biological function
Source: PeerJ. 2016 Jan 18;4:e1581. doi: 10.7717/peerj.1581 (PMC4727968; doi:10.7717/peerj.1581)

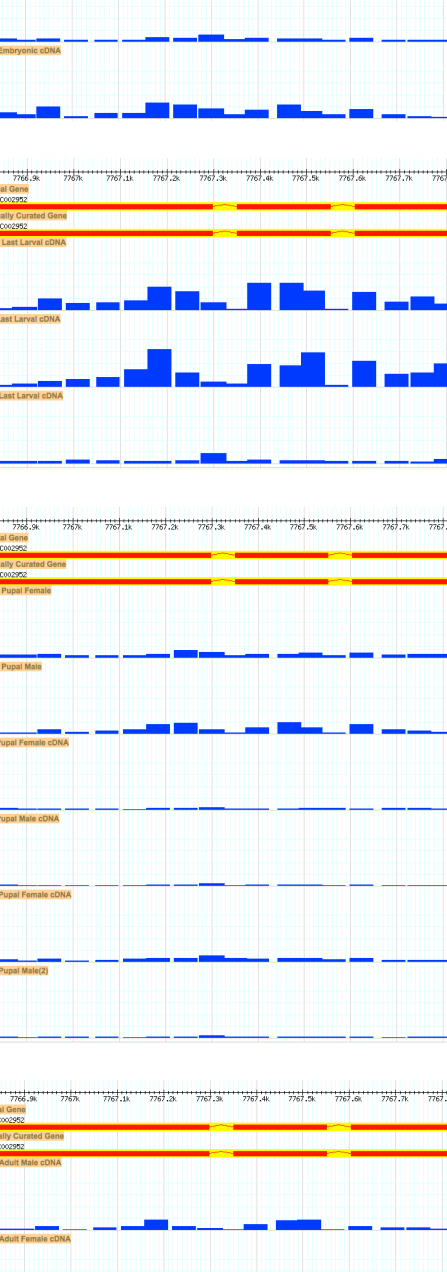

LOC663117, TC002953 (cathepsin B)

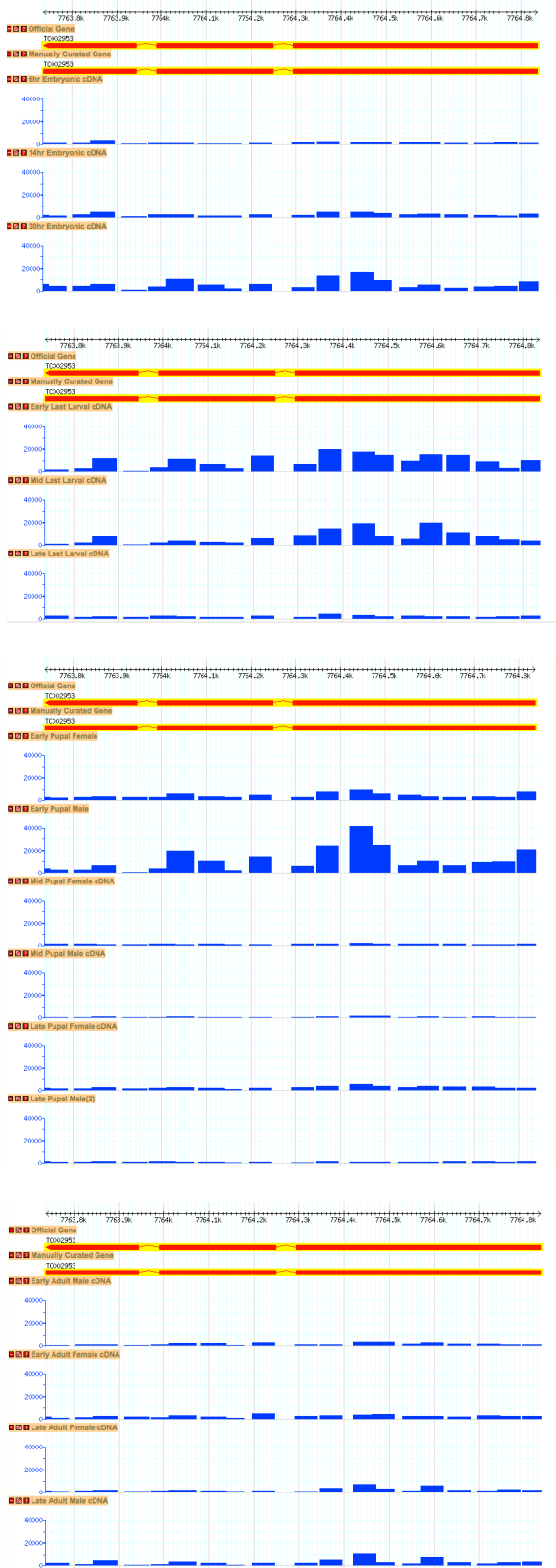

LOC663093, TC002954 (cathepsin B)

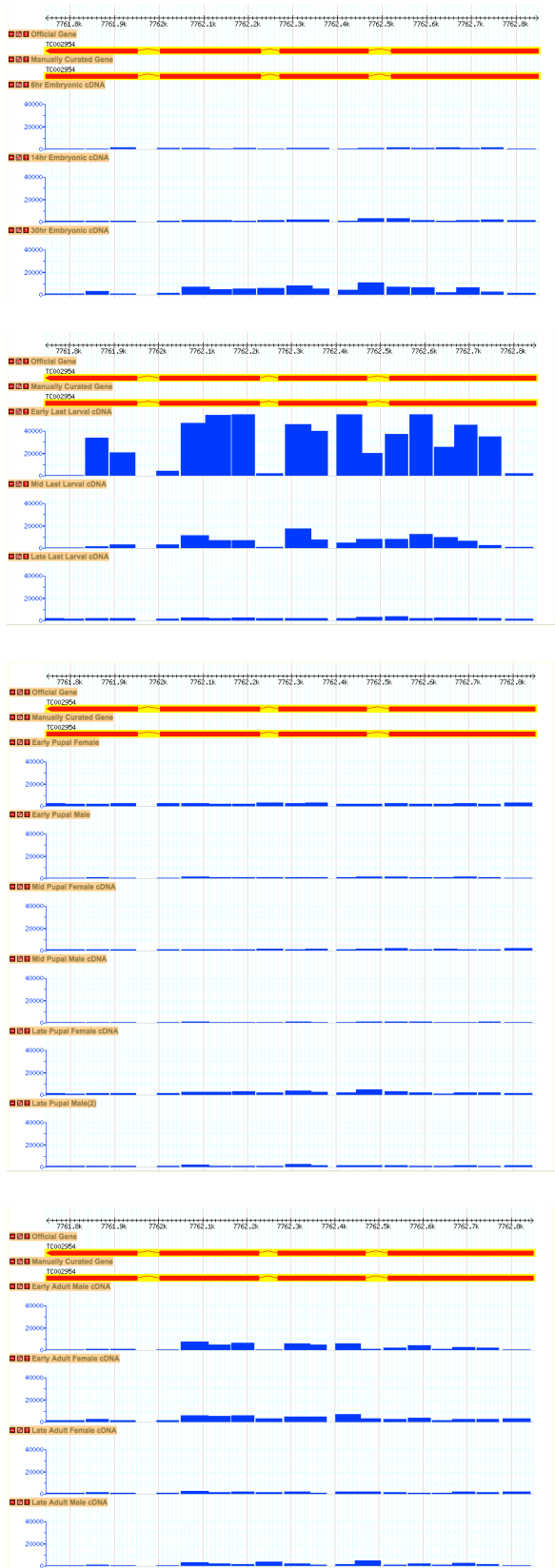

LOC663066, TC002955 (cathepsin B-like)

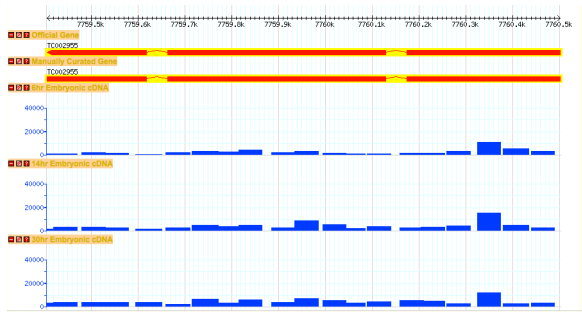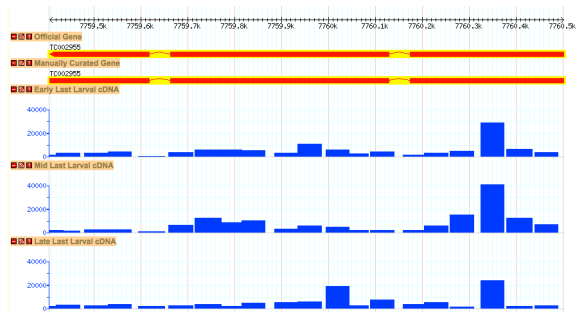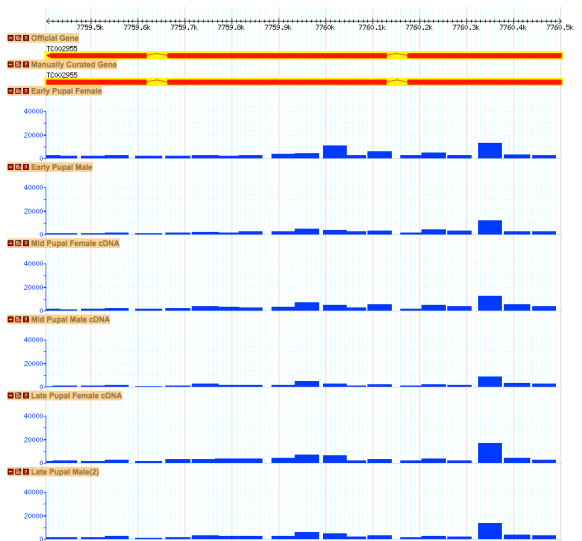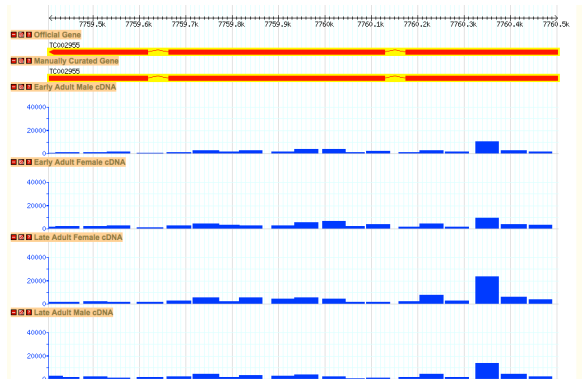

Supplement: Figure S1 — Data extracted was from: 6 h, 14 h, and 30 h embryonic; early, mid and late larval; early, mid, and late male and female pupal; early and late male and female adult. [file peerj-04-1581-s001.pdf]

Fig S2  
LOV660551, TC009362 (cathepsin L)

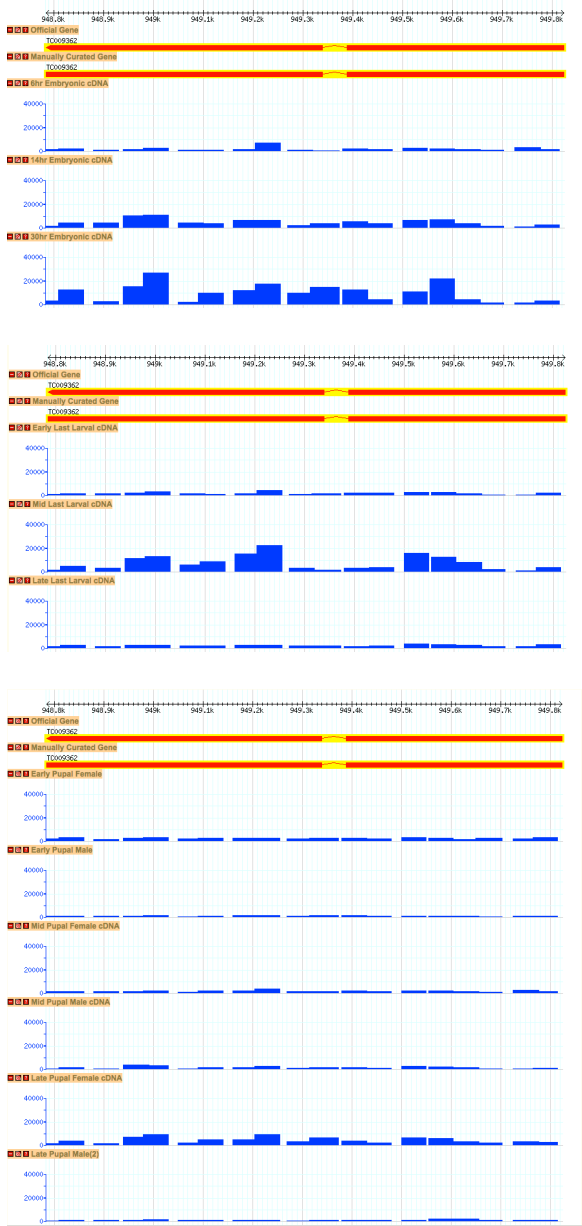

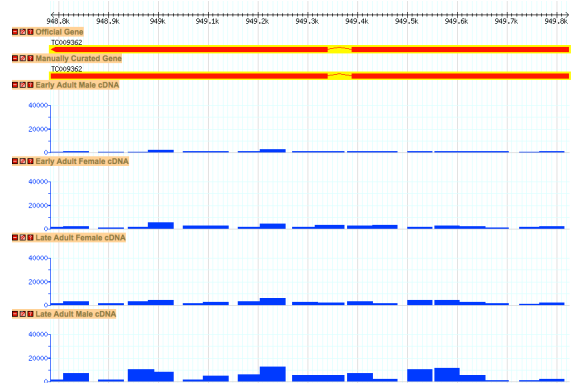

LOC660428, TC009364 (cathepsin L)

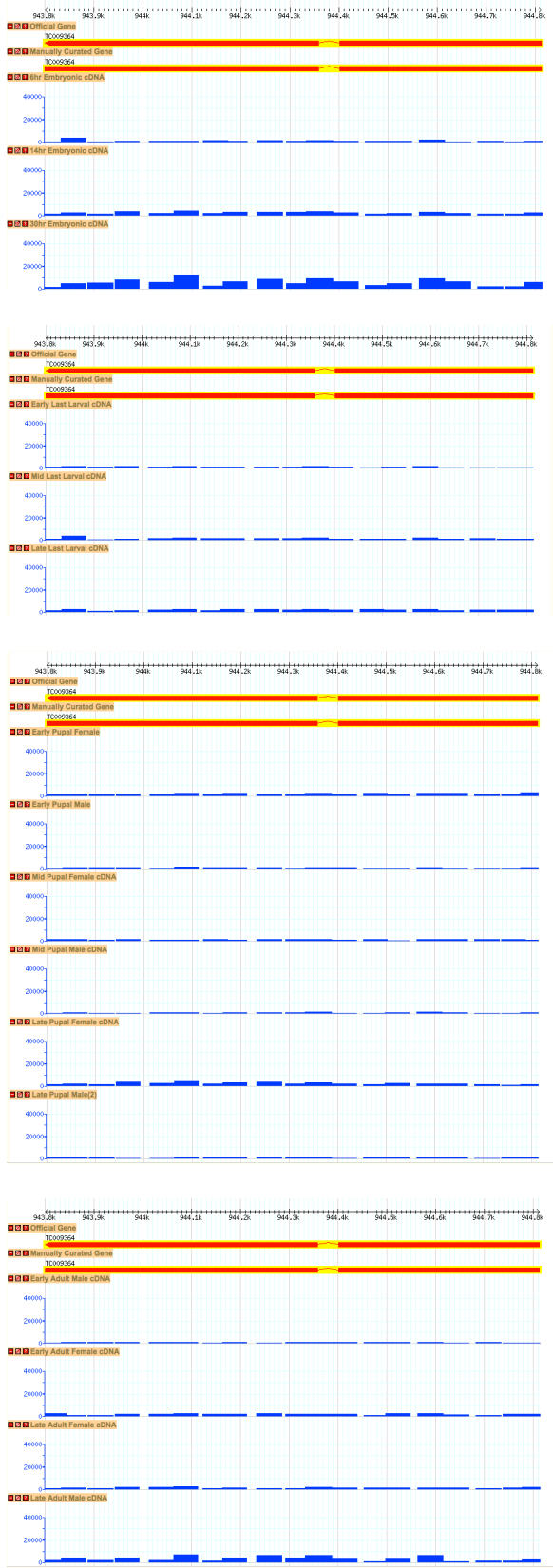

LOC660368, TC009365 (cathepsin L)

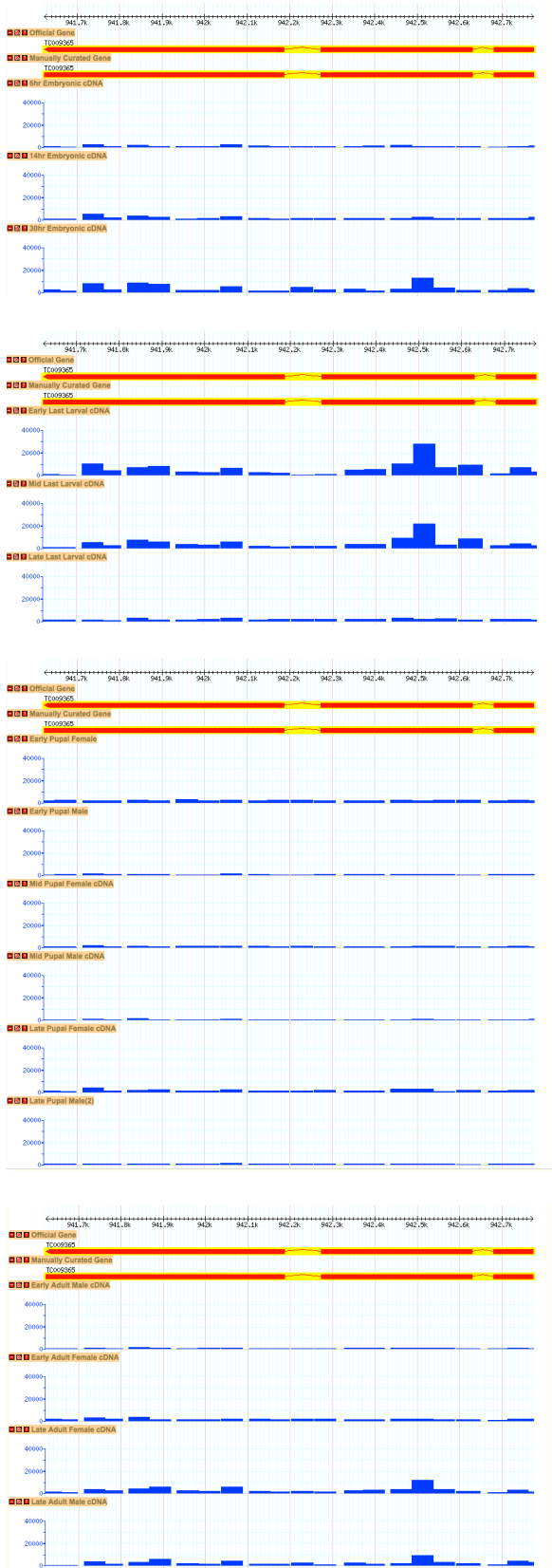

LOC660669, TC009448 (cathepsin L)

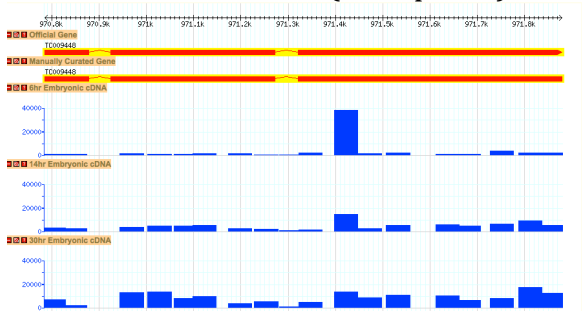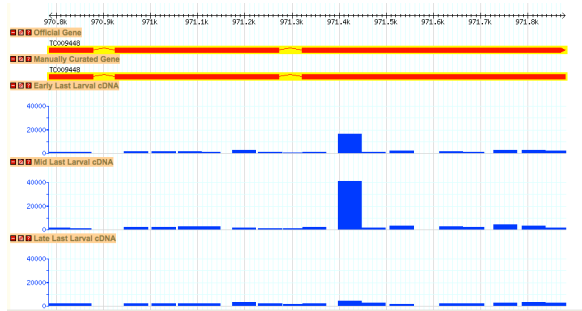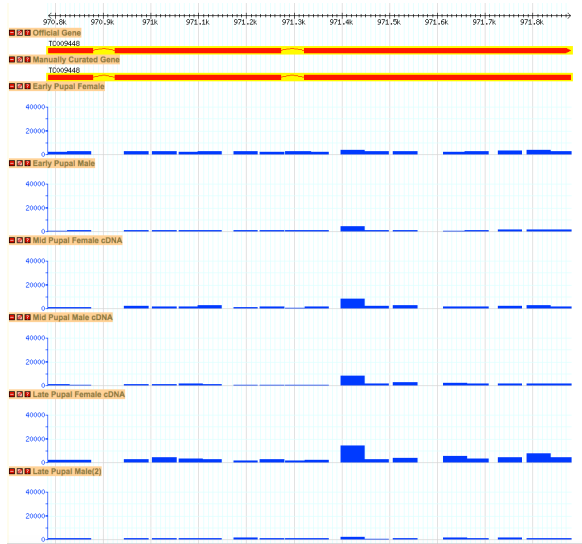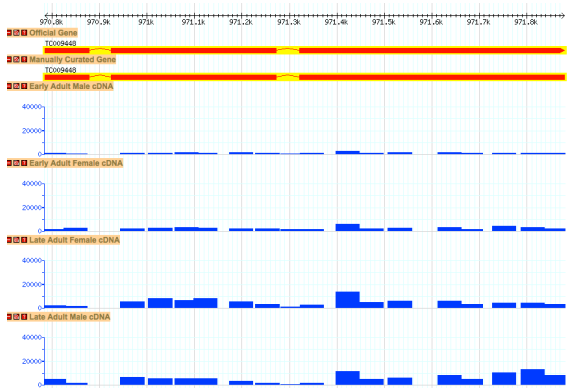

Supplement: Figure S2 — Data extracted was from: 6 h, 14 h, and 30 h embryonic; early, mid and late larval; early, mid, and late male and female pupal; early and late male and female adult. [file peerj-04-1581-s002.pdf]

FigS6

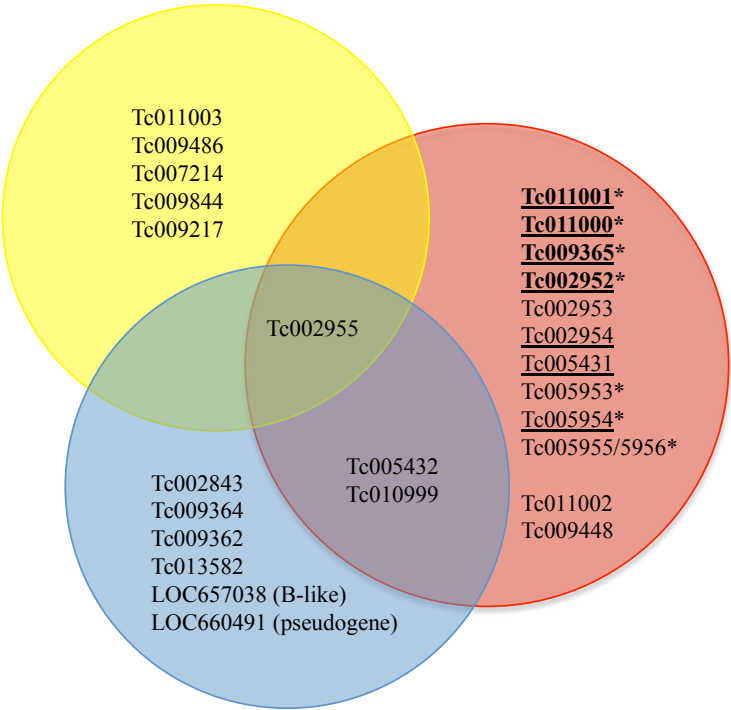

Supplement: Figure S6 — An alternate model containing TC gene numbers (i.e., GLEAN numbers from the early annotation project), which some Tribolium researchers may find easier to use. See Fig. 6 for corresponding LOC gene numbers. [file peerj-04-1581-s006.pdf]
