# Supplementary material for: Expression patterns of cysteine peptidase genes across the Tribolium castaneum life cycle provide clues to biological function
Source: PeerJ. 2016 Jan 18;4:e1581. doi: 10.7717/peerj.1581 (PMC4727968; doi:10.7717/peerj.1581)
Supplement: Figure S3 — Data extracted was from: 6 h, 14 h, and 30 h embryonic; early, mid and late larval; early, mid, and late male and female pupal; early and late male and female adult. [file peerj-04-1581-s003.pdf]

FigS3a
LOC655148, TC005431 (cathepsin B-like)

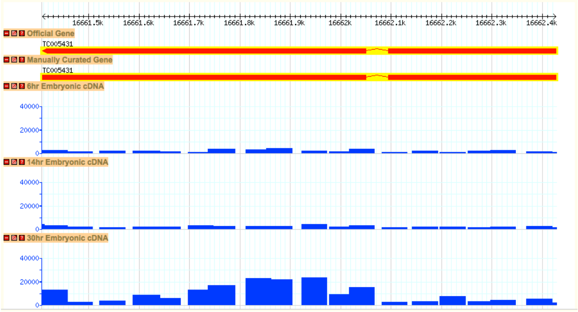

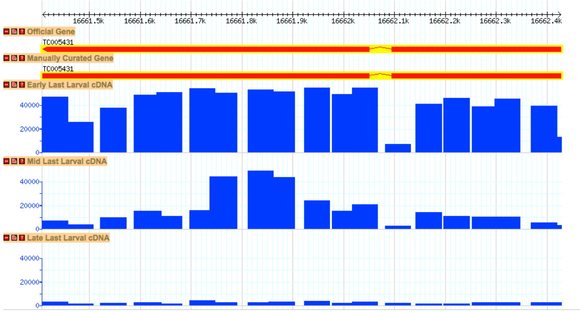

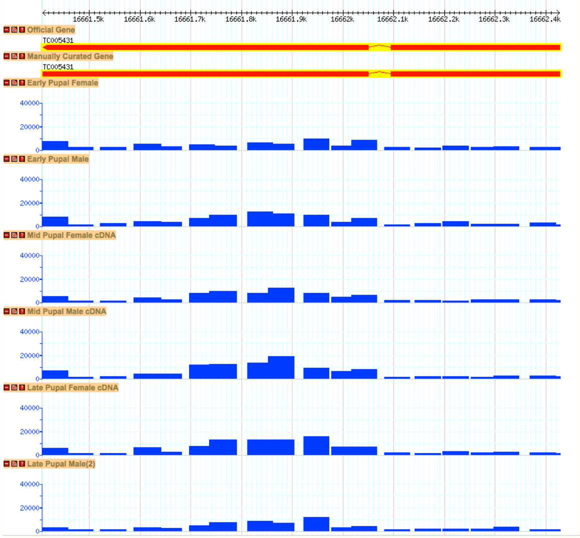

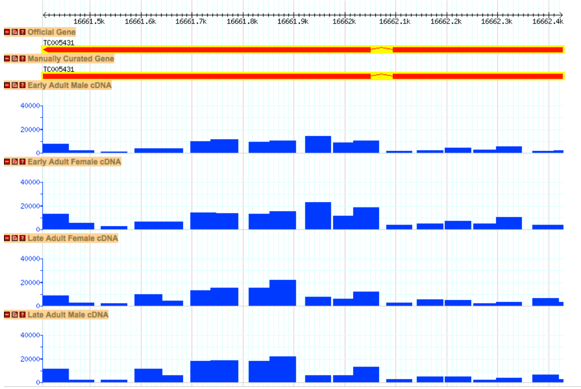
16661.5k
16661.6k
16661.7k
16661.8k
16661.9k
16662k
16662.1k
16662.2k
16662.3k
16662.4k
Official Gene
TC005431
Manually Curated Gene
TC005431
Early Adult Male cDNA
40000
20000
0
Early Adult Female cDNA
40000
20000
0
Late Adult Female cDNA
40000
20000
0
Late Adult Male cDNA
40000
20000
0

LOC655077, TC005432 (cathepsin B-like)

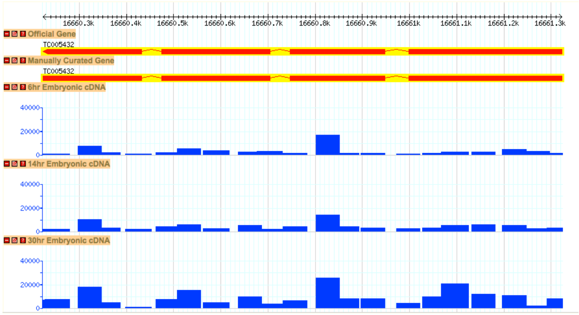

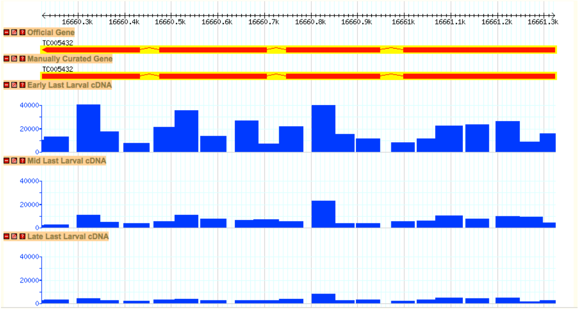

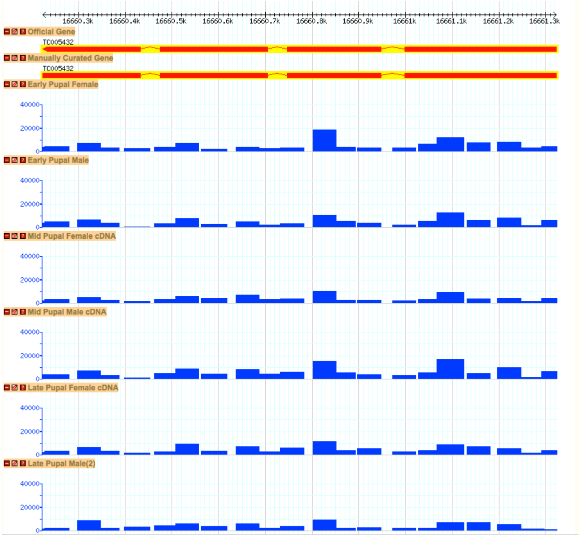

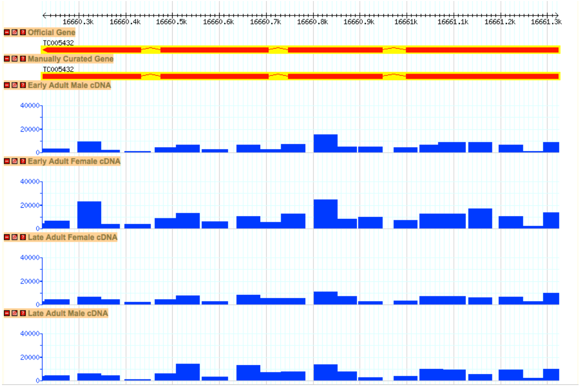

FigS3b

LOC657203, TC005953 (cathepsin B-like)

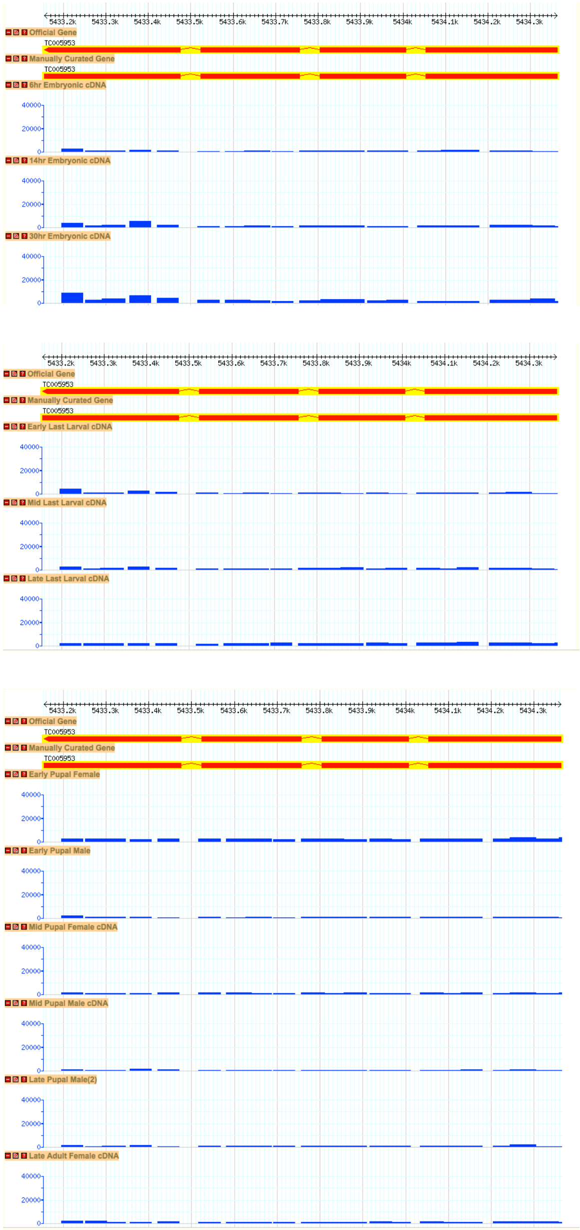

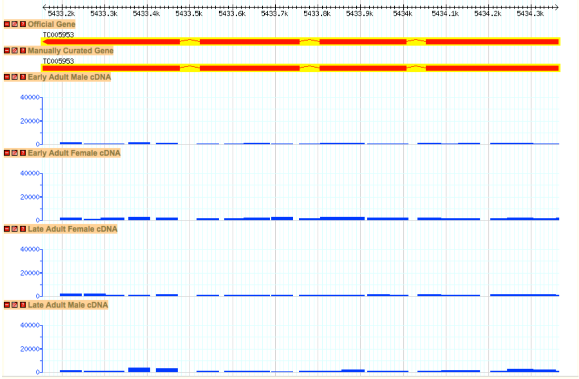
5433.2k
5433.3k
5433.4k
5433.5k
5433.6k
5433.7k
5433.8k
5433.9k
5434k
5434.1k
5434.2k
5434.3k
Official Gene
TC005953
Manually Curated Gene
TC005953
Early Adult Male cDNA
40000
20000
0
Early Adult Female cDNA
40000
20000
0
Late Adult Female cDNA
40000
20000
0
Late Adult Male cDNA
40000
20000
0

LOC657117, TC005954 (cathepsin B homolog)

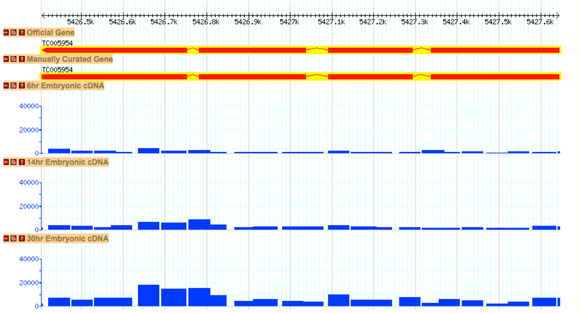

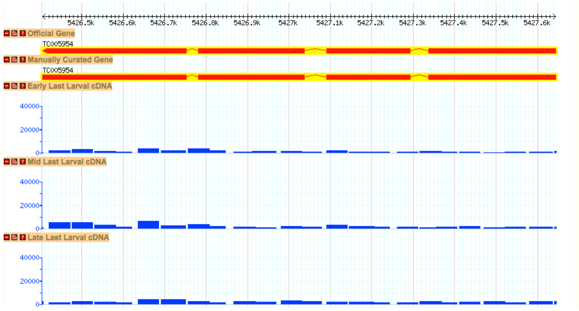

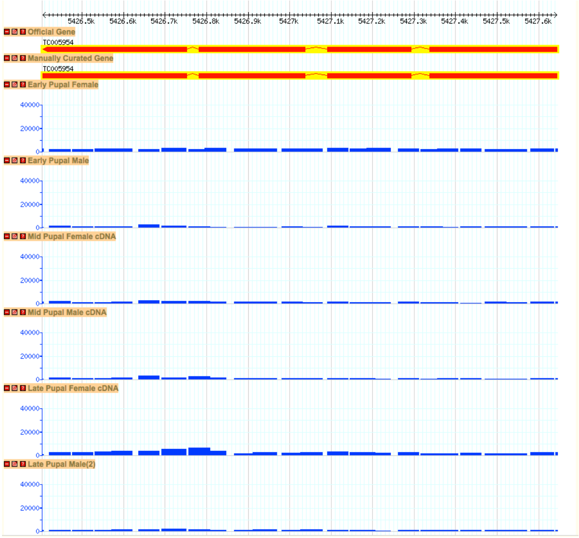

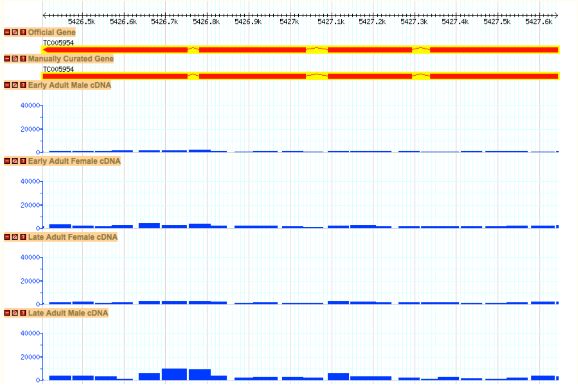

LOC656957, TC005955/5956* (cathepsin B-like)

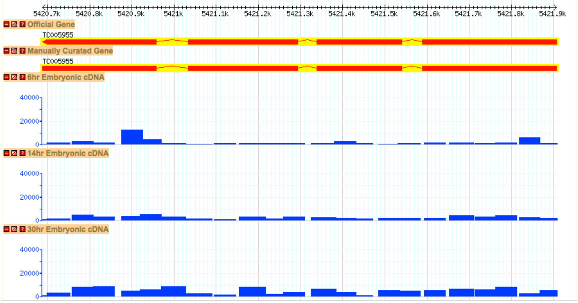

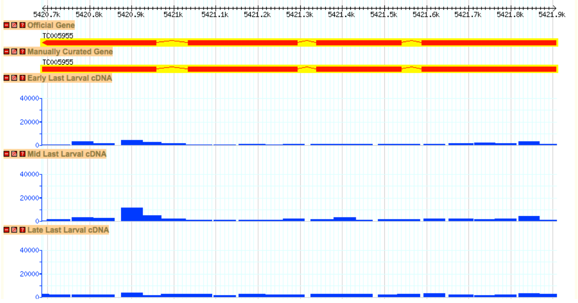

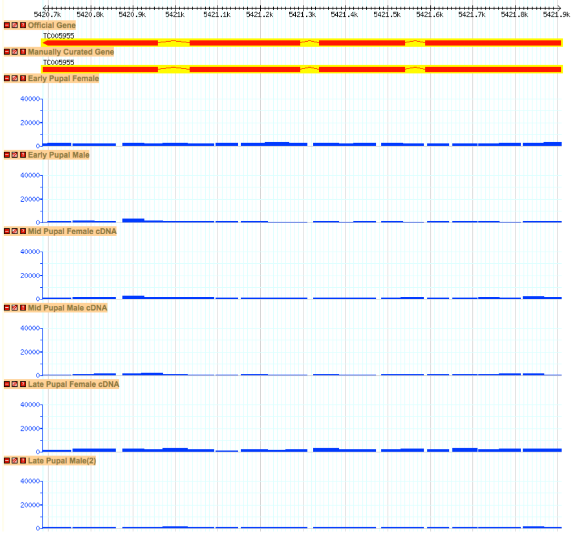

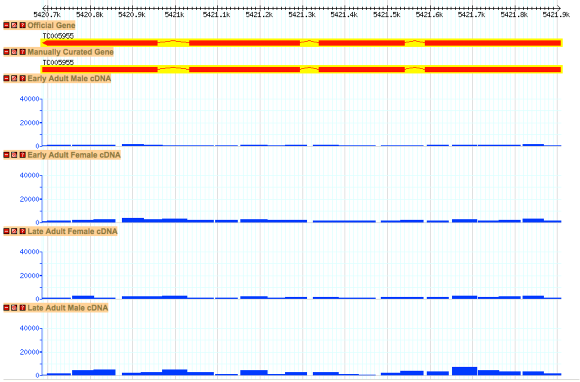

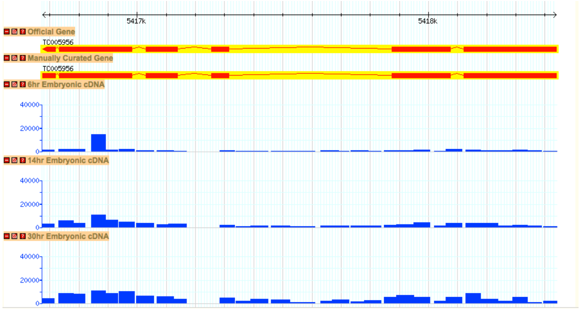

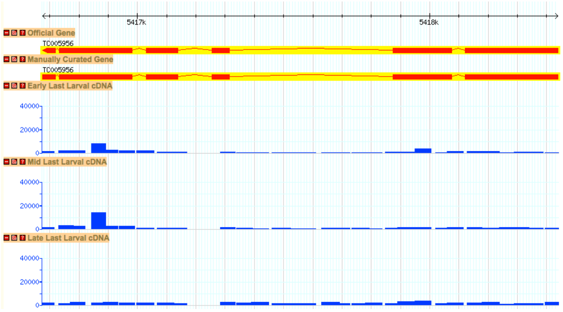

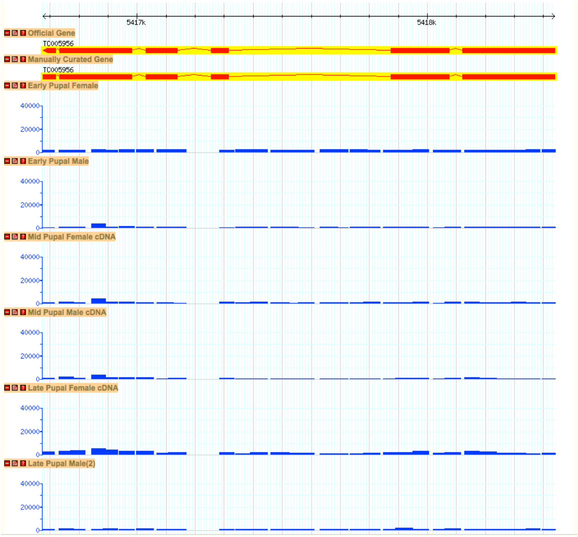

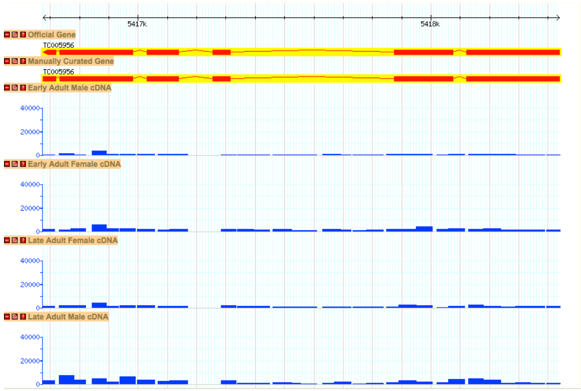

*Note: While we have annotated these as identical genes (Martynov et al., 2012) and according to TCas4.0 in NCBI, these were tiled separately in Beetlebase.
