# Supplementary material for: Expression patterns of cysteine peptidase genes across the Tribolium castaneum life cycle provide clues to biological function
Source: PeerJ. 2016 Jan 18;4:e1581. doi: 10.7717/peerj.1581 (PMC4727968; doi:10.7717/peerj.1581)

FigS4
LOC659565, TC010999 (cathepsin L)

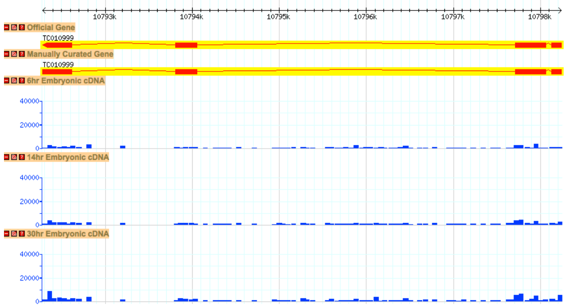

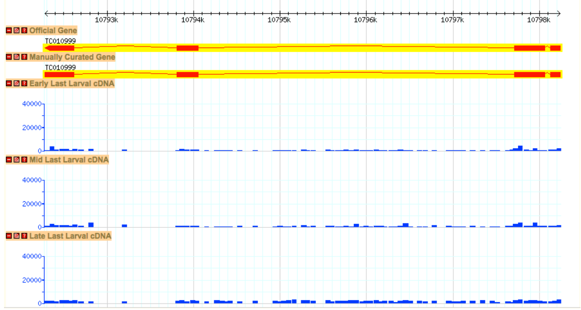

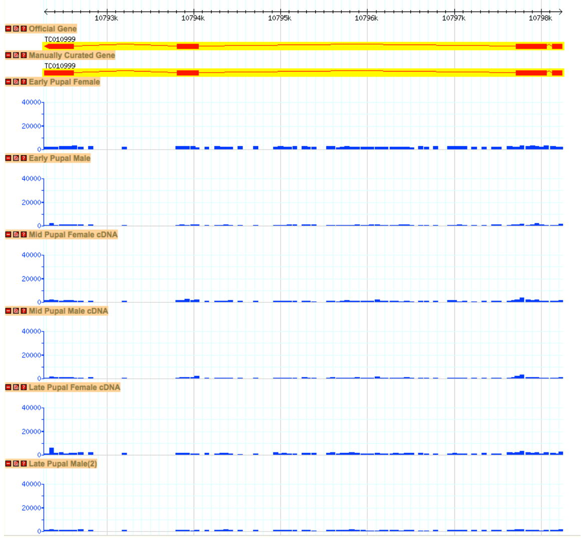

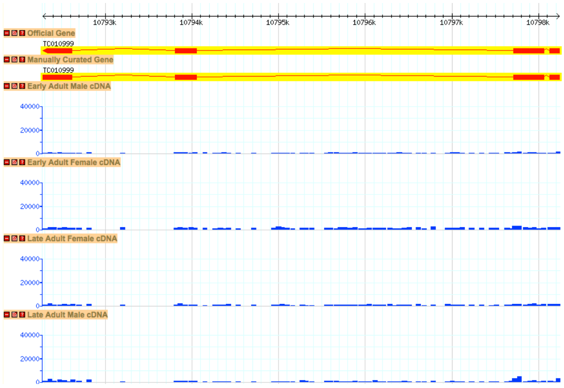

10793k
10794k
10795k
10796k
10797k
10798k
Official Gene
TC010999
Manually Curated Gene
TC010999
Early Adult Male cDNA
Early Adult Female cDNA
Late Adult Female cDNA
Late Adult Male cDNA

LOC659502, TC011000 (cathepsin L)

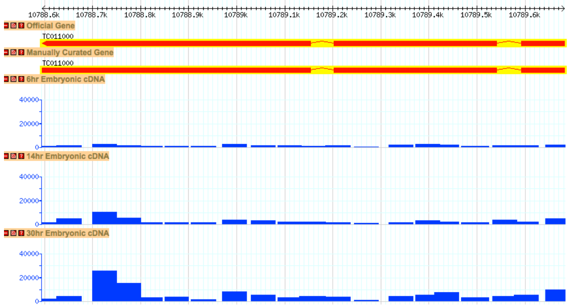

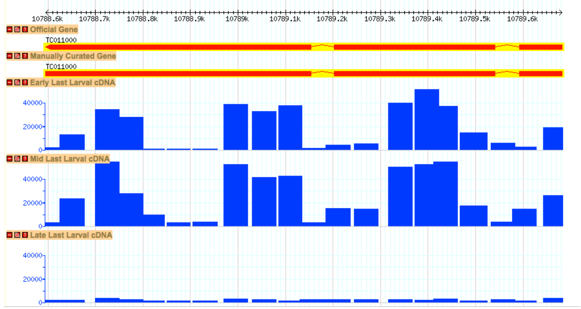

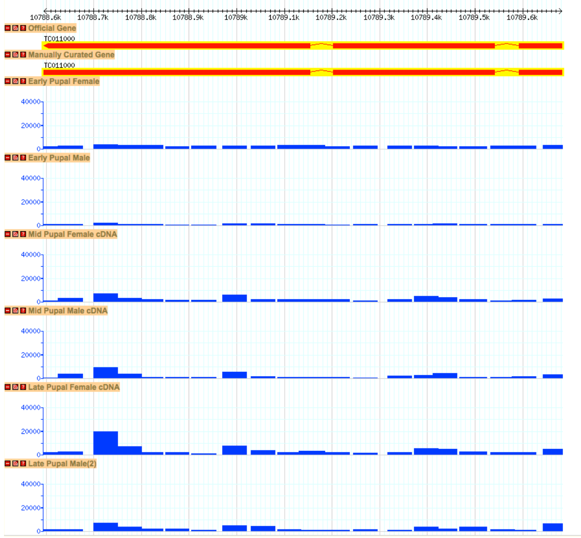

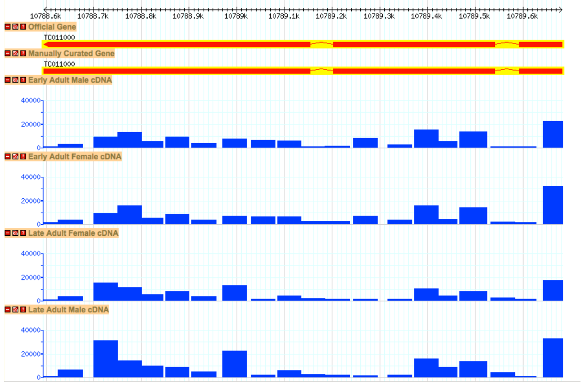

LOC659441, TC011001 (cathepsin L)

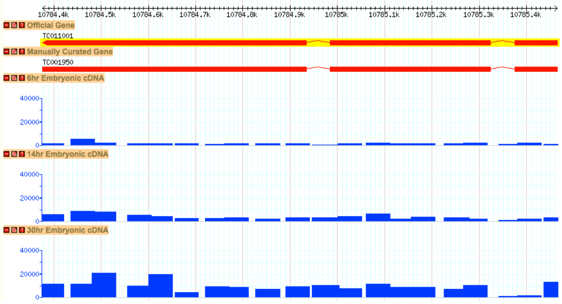

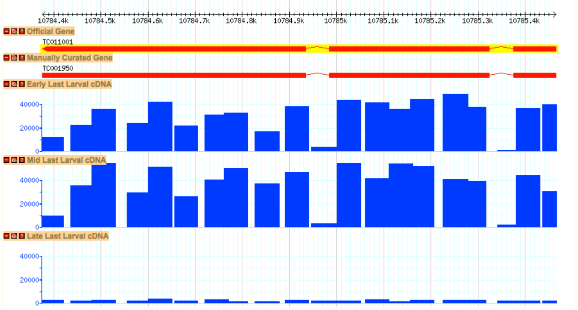

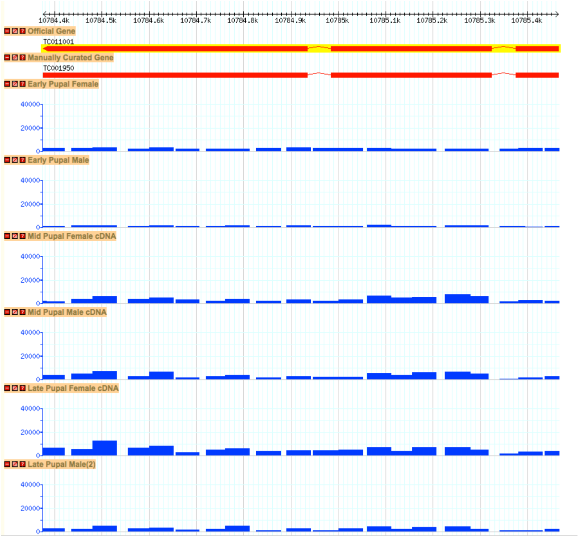

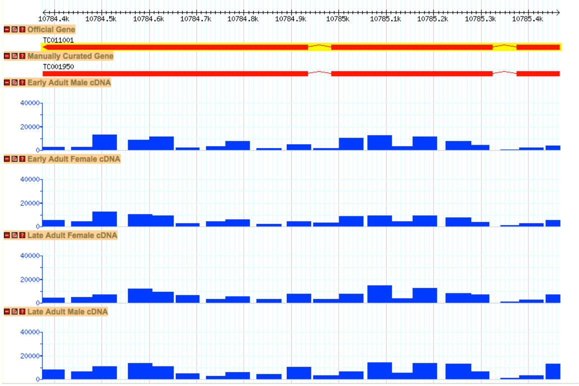

LOC659367, TC011002 (cathepsin L homolog)

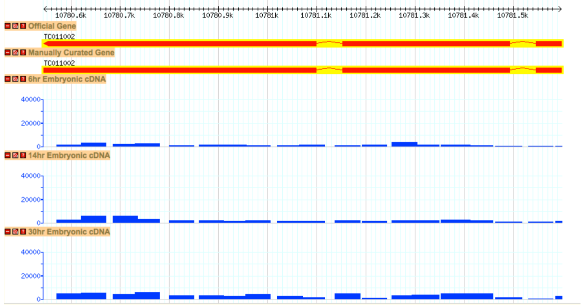

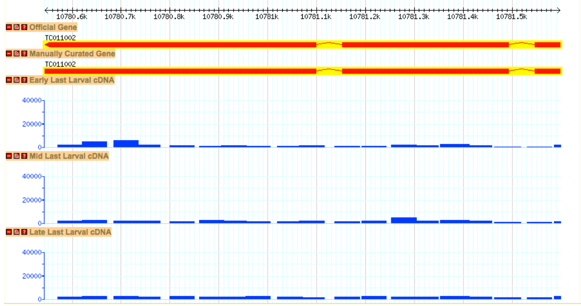

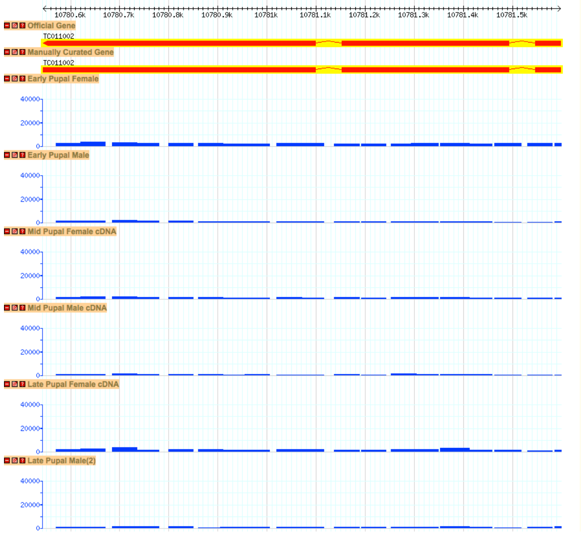

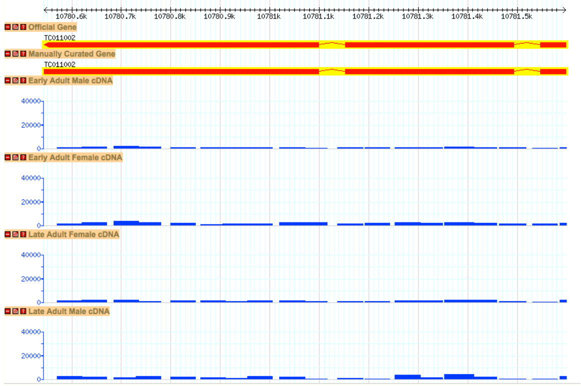

LOC659367, TC011003 (cathepsin L)

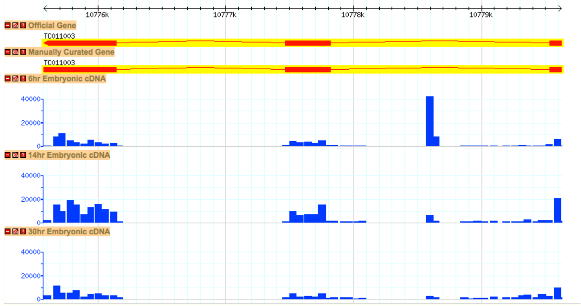

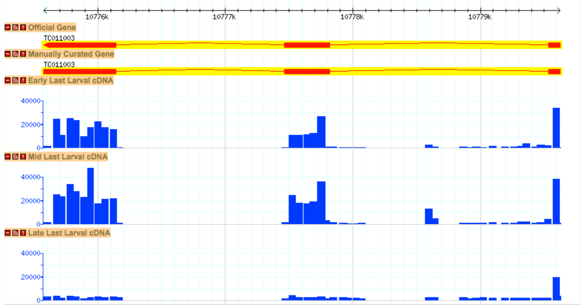

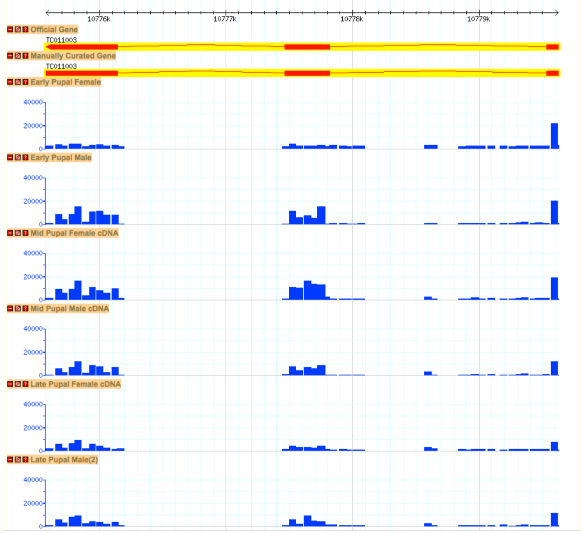

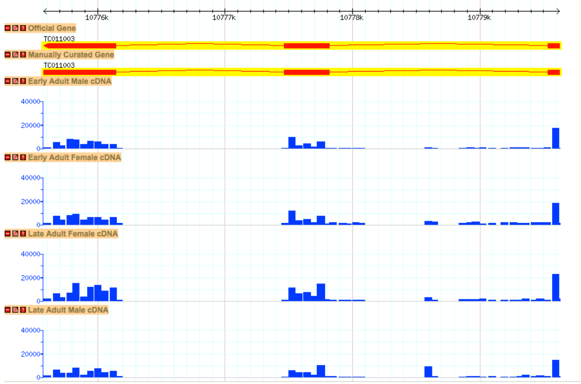

Supplement: Figure S4 — Data extracted was from: 6 h, 14 h, and 30 h embryonic; early, mid and late larval; early, mid, and late male and female pupal; early and late male and female adult. [file peerj-04-1581-s004.pdf]
