# Supplementary material for: Expression patterns of cysteine peptidase genes across the Tribolium castaneum life cycle provide clues to biological function
Source: PeerJ. 2016 Jan 18;4:e1581. doi: 10.7717/peerj.1581 (PMC4727968; doi:10.7717/peerj.1581)

FigS5

LOC663234, TC009486 (cathepsin L)

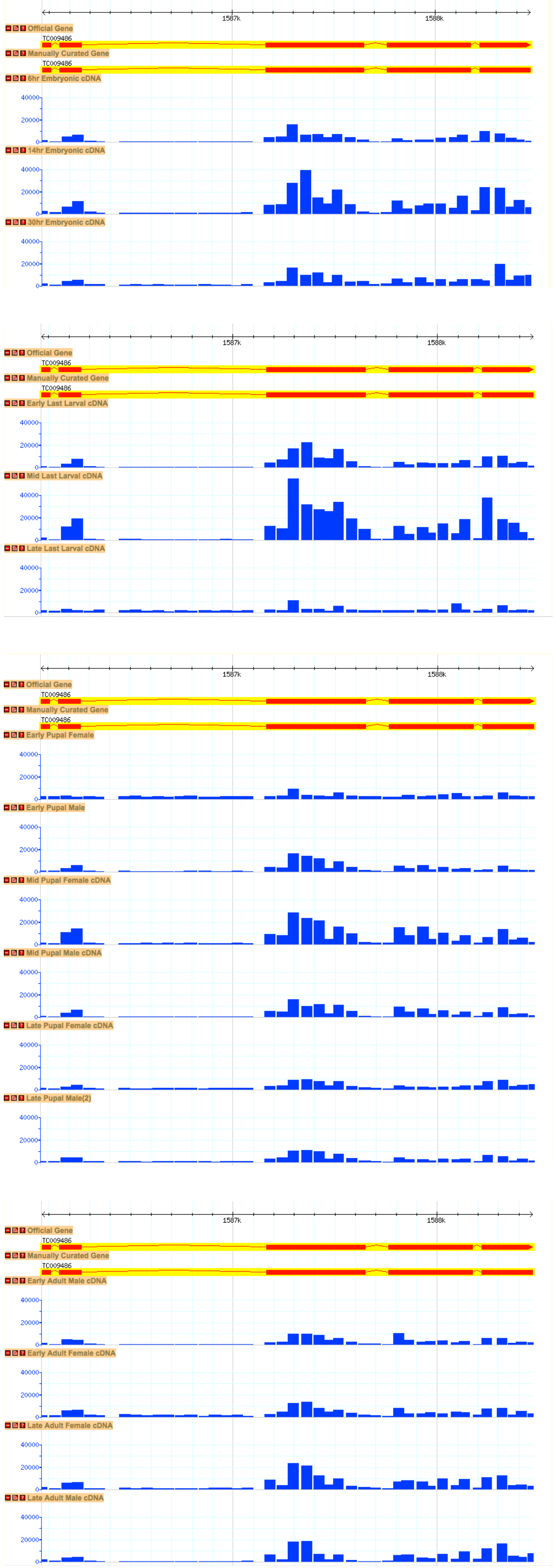

LOC658343, TC002843 (cathepsin L)

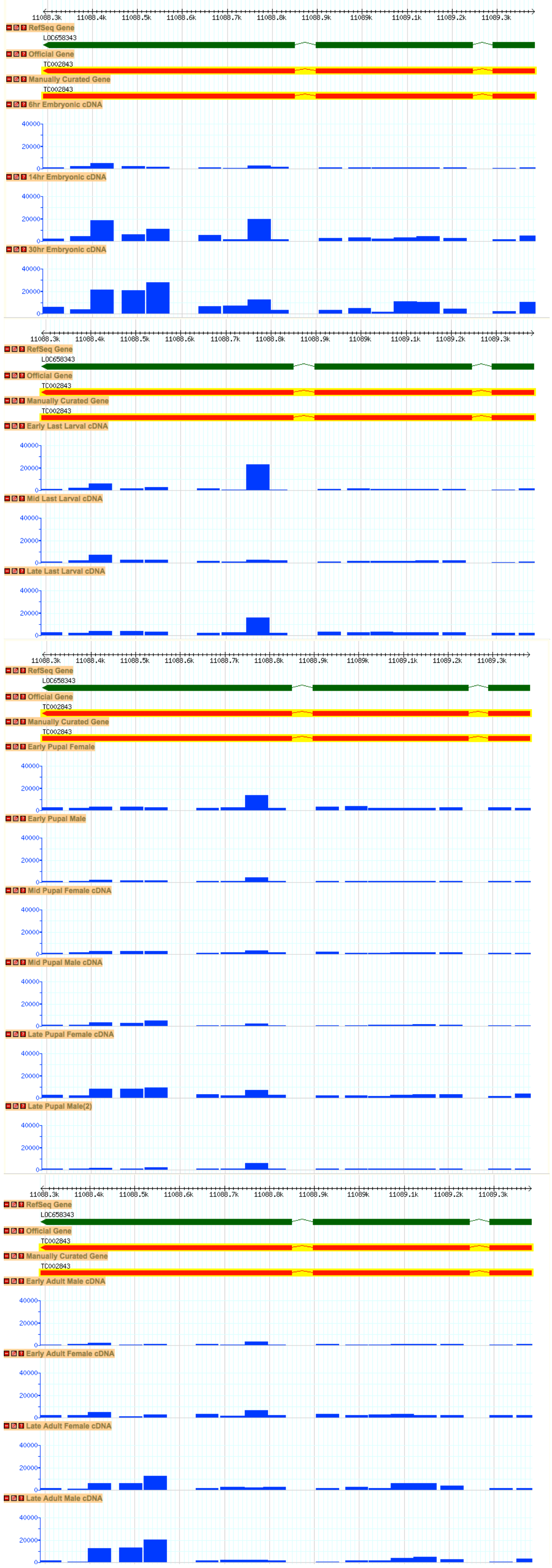

LOC656198, TC009217 (cathepsin B homolog)

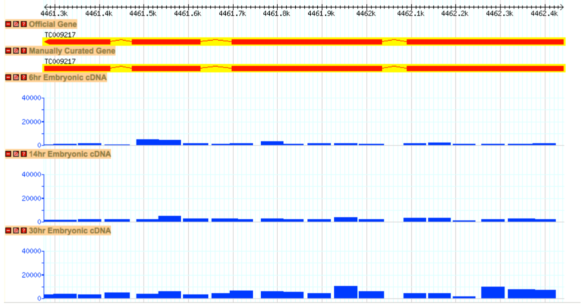

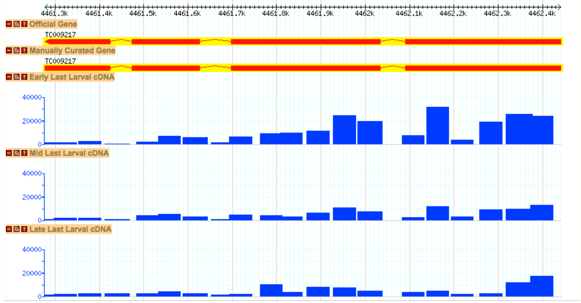

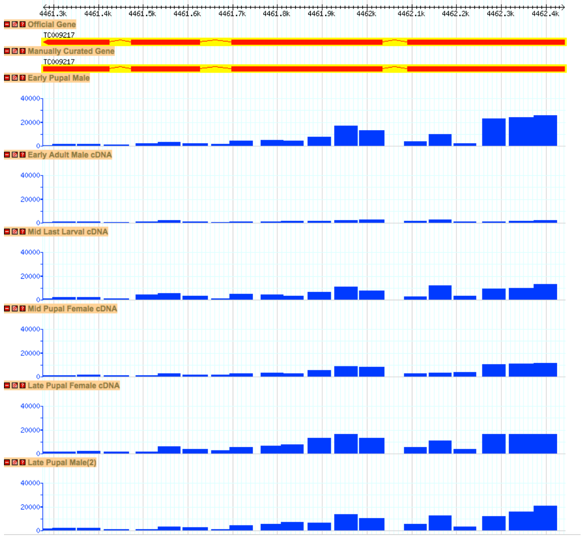

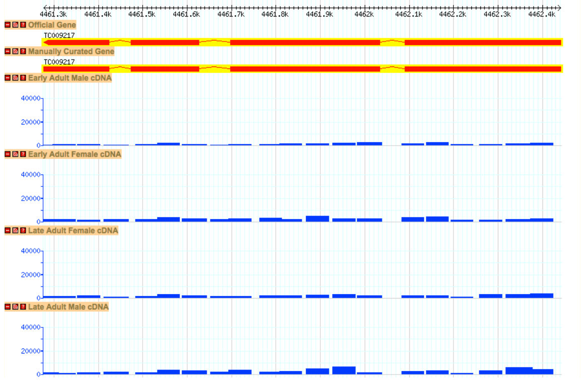

LOC659087, TC007214 (cathepsin O)

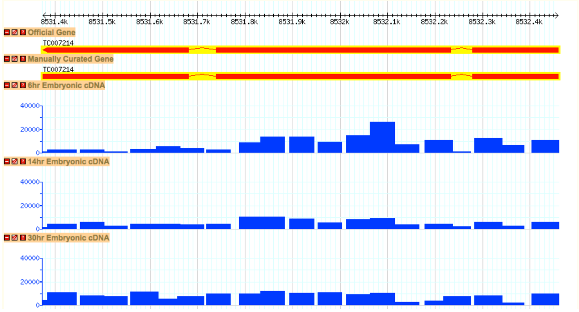

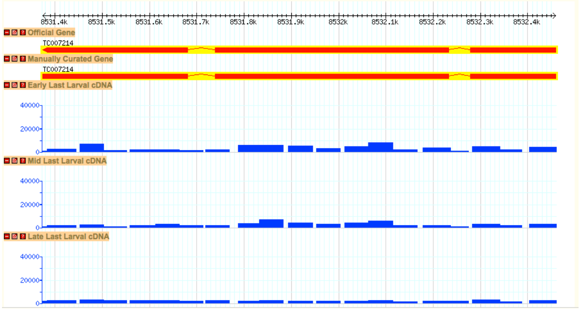

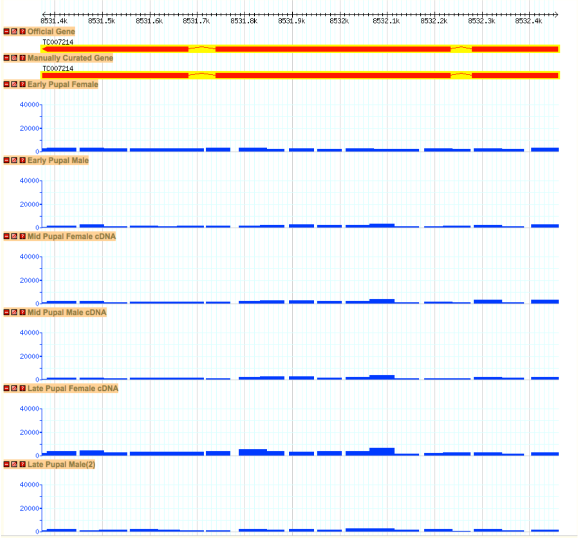

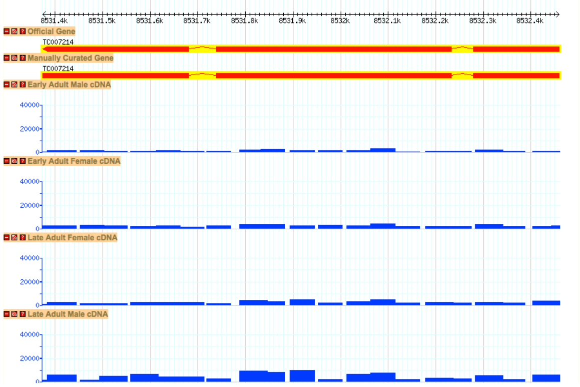

LOC662417 (cathepsin F)

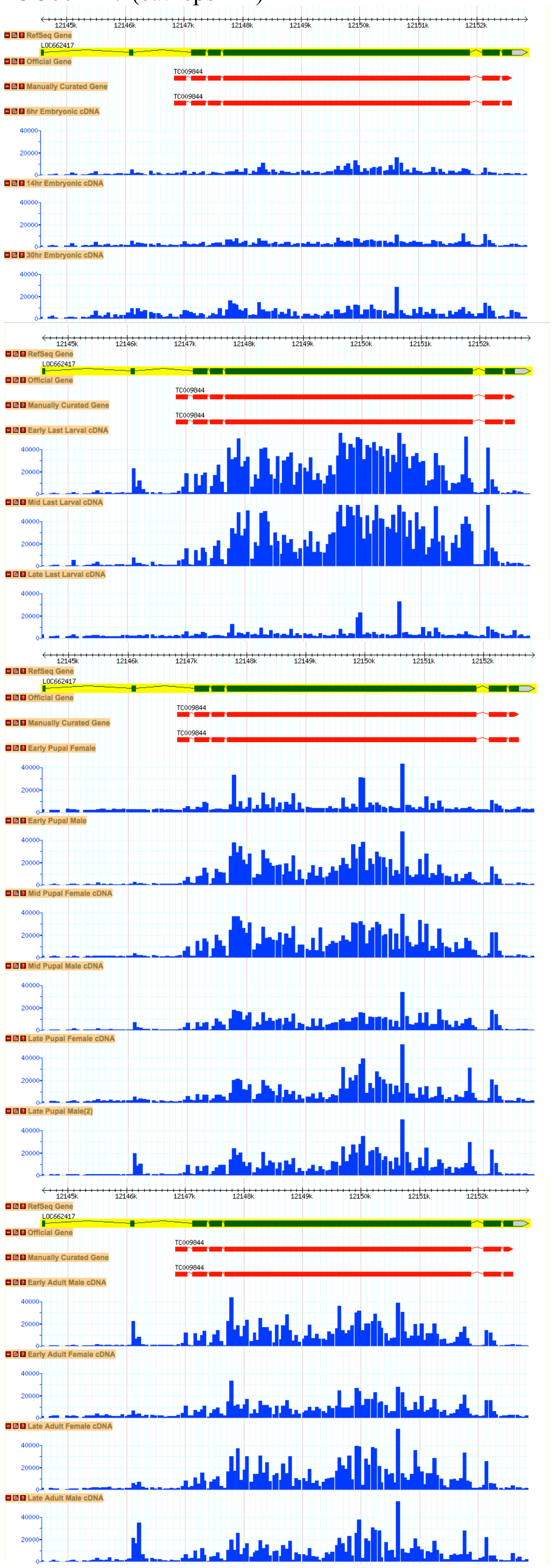

LOC100141668, TC013582 (cathepsin K)

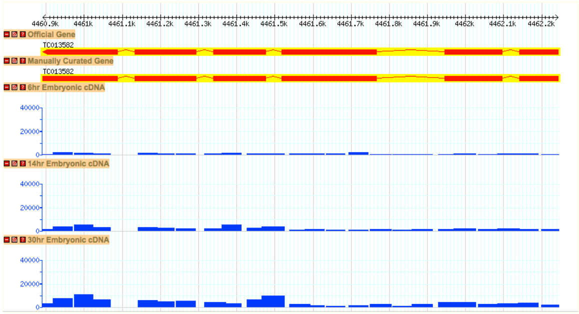

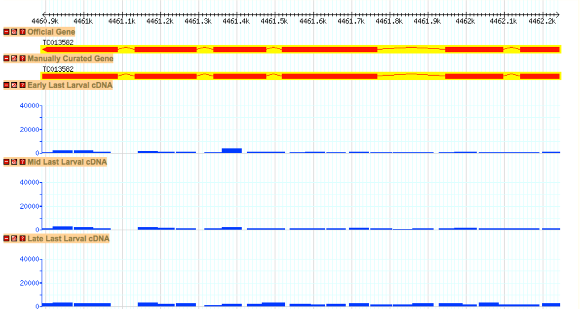

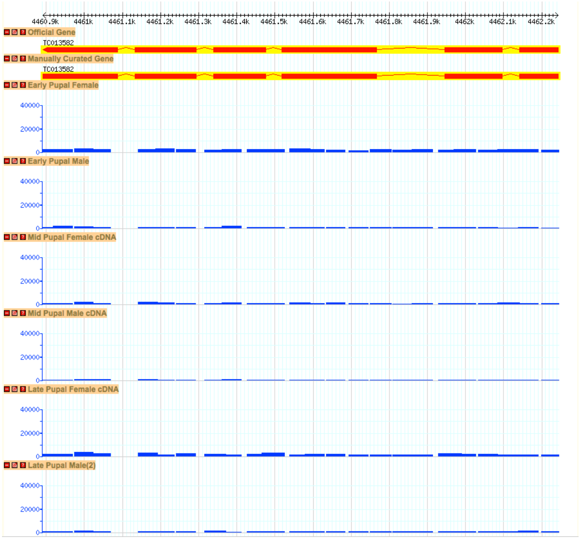

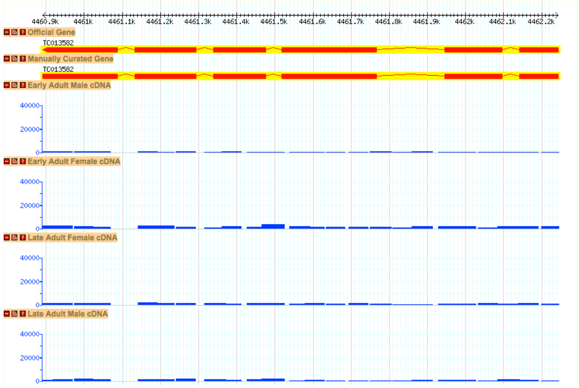

Supplement: Figure S5 — Data extracted was from: 6 h, 14 h, and 30 h embryonic; early, mid and late larval; early, mid, and late male and female pupal; early and late male and female adult. [file peerj-04-1581-s005.pdf]
