# Supplementary material for: Expression patterns of cysteine peptidase genes across the Tribolium castaneum life cycle provide clues to biological function
Source: PeerJ. 2016 Jan 18;4:e1581. doi: 10.7717/peerj.1581 (PMC4727968; doi:10.7717/peerj.1581)
Supplement: Table S1 — General information on sequencing runs for each biological replicate of each life stage. Total reads refers to the number of useable reads. [file peerj-04-1581-s007.pdf]

Supplemental Table S1. General information on sequencing runs for each biological replicate of each life stage. Total reads refers to the number of useable reads.

Biological sample	Replicate	Total reads	Mean read length (bp)
Egg	1	2,317,749	88
Egg	2	3,591,830	97
Egg	3	1,429,150	56
Larvae	1	1,182,910	46
Larvae	2	2,263,982	81
Larvae	3	2,063,167	77
Pupae	1	5,299,156	111
Pupae	2	4,772,227	114
Pupae	3	3,834,655	84
Adult	1	2,781,808	91
Adult	2	2,245,771	81
Adult	3	4,908,253	71
