# Supplementary material for: Expression patterns of cysteine peptidase genes across the Tribolium castaneum life cycle provide clues to biological function
Source: PeerJ. 2016 Jan 18;4:e1581. doi: 10.7717/peerj.1581 (PMC4727968; doi:10.7717/peerj.1581)
Supplement: Table S2 — The mean linear total RPKM values for each cysteine peptidase gene in each T. castaneum life stage with standard error for each mean. P-value is from an Analysis of Variance (ANOVA) of expression values (RPKM) across life stages after multiple testing correction (FDR) and before correction (none). [file peerj-04-1581-s008.pdf]

Supplemental Table S2. The mean linear total RPKM values for each cysteine peptidase gene in each *T. castaneum* life stage with standard error for each mean. P-value is from Kruskal-Wallis test (df=3).

	RPKM (mean linear)				Standard error				Kruskal-Wallis	
**Gene ID**	**Adult**	**Egg**	**Larvae**	**Pupae**	**Adult**	**Egg**	**Larvae**	**Pupae**	**χ2**	**p-value**
LOC659441	3,850	0.50	55,600	11.2	0.56	2.11	0.21	4.33	9.21	0.03
LOC659502	6,390	0.03	13,600	1.90	0.57	0.00	0.09	3.05	9.21	0.03
LOC659226	817	734	878	26.4	0.42	0.19	0.08	4.95	1.51	0.68
LOC659367	42.9	0.03	24.0	0.03	0.77	0.00	0.16	0.00	10.2	0.02
LOC659565	3.82	5.75	0.15	1.54	0.97	0.69	2.40	2.91	3.55	0.31
LOC660368	489	0.16	1,870	0.03	0.86	2.56	0.12	0.00	9.07	0.03
LOC660551	0.61	0.03	0.42	0.03	2.37	0.00	1.97	0.00	5.41	0.14
LOC660428	8.29	0.13	0.03	0.03	0.78	2.25	0.00	0.00	9.31	0.03
LOC660669	7.77	0.10	0.89	0.08	0.91	1.90	2.56	1.57	8.42	0.04
LOC663234	233	324	287	818	0.11	0.28	0.17	0.20	3.00	0.39
LOC660491	0.03	0.03	0.03	0.03	0.00	0.00	0.00	0.00	-	-
LOC656198	35.9	25.2	44.5	4.04	0.90	0.54	0.19	3.60	1.51	0.68
LOC662417	57.9	18.7	42.7	5.30	0.33	0.11	0.08	3.80	3.82	0.28
LOC663145	669	0.57	1,690	1.47	0.24	2.40	0.20	2.86	8.56	0.04
LOC663117	65.6	0.03	162	4.42	0.16	0.03	0.11	3.66	5.86	0.12
LOC663090	93.3	0.12	148	0.54	0.52	1.96	0.12	2.17	9.08	0.03

LOC663066	337	188	372	14.6	0.28	0.07	0.08	4.52	2.99	0.41
LOC658343	0.36	0.10	0.03	0.03	1.85	1.90	0.00	0.00	-	-
LOC655148	112	0.13	59.5	0.03	1.02	2.25	0.17	0.00	9.40	0.02
LOC655077	0.10	0.03	0.03	0.03	1.82	0.00	0.00	0.00	-	-
LOC657117	9.14	0.03	1.65	0.33	0.60	0.00	2.96	1.85	7.19	0.07
LOC657203	4.16	0.03	10.8	0.03	0.68	0.00	0.42	0.00	9.50	0.02
LOC656957	9.87	0.03	0.64	0.08	0.812	0.00	2.40	1.48	8.42	0.04
LOC657038	0.03	0.03	0.03	0.03	0.00	0.00	0.00	0.00	-	-
LOC659087	36.1	35.3	15.4	115	0.37	0.07	0.15	0.26	8.08	0.04
LOC100141668	1.24	0.03	0.10	0.03	2.75	0.00	1.88	0.00	4.99	0.17
