# Supplementary material for: Expression patterns of cysteine peptidase genes across the Tribolium castaneum life cycle provide clues to biological function
Source: PeerJ. 2016 Jan 18;4:e1581. doi: 10.7717/peerj.1581 (PMC4727968; doi:10.7717/peerj.1581)
Supplement: Table S3 — Data is the percent of individuals with the defined mortality/morphology out of 10 individuals. Blank boxes indicate information was not available. Primers are those used in the RNAi screen (http://ibeetle-base.uni-goettingen.de; Dönitz et al., 2015). [file peerj-04-1581-s009.pdf]

Supplemental Table S3. iBeetle phenotypes. Data is the percent of individuals with the defined mortality/morphology out of 10 individuals. Blank boxes indicate information was not available. Primers are those used in the RNAi screen (http://ibeetle-base.uni-goettingen.de; Dönitz et al., 2015).

LOC663066 B-like Primers: left: GGCAGAAACCGGAATTGTAA right: GCTCTCAATCCCACATTCGT

	Phenotype after larval injection				Phenotype after pupal injection			
	Egg	Larvae	Pupae	Adult	Egg	Larvae	Pupae	Adult
Mortality 11 days	-	0%	0%	0%	-	-	40%	40%
Mortality 22 days	-	0%	0%	0%				
Fertility								
Morphology								

LOC656957 B-like Primers: left: TGTGGGTGTAAAGGGGGTTA right: TTCTTCTGTTCCCCACCCTA

	Phenotype after larval injection				Phenotype after pupal injection			
	Egg	Larvae	Pupae	Adult	Egg	Larvae	Pupae	Adult
Mortality 11 days	-	10%	10%	10%	-	-	0%	0%
Mortality 22 days	-	10%	10%	10%				
Fertility								
Morphology								

LOC656198 B homolog Primers: left: GCCAATTATGGATGGAGTGC right: GGTCAACATTGGTCGGAAGT

	Phenotype after larval injection				Phenotype after pupal injection			
	Egg	Larvae	Pupae	Adult	Egg	Larvae	Pupae	Adult
Mortality 11 days	-	0%	0%	0%			0%	0%
Mortality 22 days	-	20%	20%	20%				
Fertility								
Morphology				Eclosion not fulfilled	No cuticle (30%)	Irregular musculature (50-80%)		

LOC657117 B homolog Primers: left: CAAGCGACCCAAAACGTAAT right: ATAGACATCGAAGGCGGCTA

	Phenotype after larval injection				Phenotype after pupal injection			
	Egg	Larvae	Pupae	Adult	Egg	Larvae	Pupae	Adult
Mortality 11 days	-	0%	0%	0%	-	-	10%	10%
Mortality 22 days	-	0%	0%	0%	-	-		
Fertility								
Morphology								Eclosion not fulfilled

LOC663145 B Primers: left: GGCAGAAACCGGAATTGTAA right: GCTCTCAATCCCACATTCGT

	Phenotype after larval injection				Phenotype after pupal injection			
	Egg	Larvae	Pupae	Adult	Egg	Larvae	Pupae	Adult
Mortality 11 days	-	0%	0%	0%	-	-	10%	10%
Mortality 22 days	-	0%	0%	0%	-	-		
Fertility								
Morphology					Without cuticle (30-50%)			

LOC663090 B Primers: left: GATACATGATGGCTGCGTTC right: CAATCCCACATTCATTTTTGC

	Phenotype after larval injection				Phenotype after pupal injection			
	Egg	Larvae	Pupae	Adult	Egg	Larvae	Pupae	Adult
Mortality 11 days	-	0%	0%	0%	-	-	0%	0%
Mortality 22 days	-	0%	0%	0%	-	-		
Fertility								
Morphology								

LOC660368 L Primers: left: AGTACTGGCGCCATTGAATC right: CCCTTATTCCTGGCGATTTT

	Phenotype after larval injection				Phenotype after pupal injection			
	Egg	Larvae	Pupae	Adult	Egg	Larvae	Pupae	Adult
Mortality 11 days	-	100%	100%		-	-	10%	10%
Mortality 22 days	-	100%	100%	100%	-	-		
Fertility								Vitellarium thinner (40%) Vitellogenic egg chamber not present (40%) Previtellogenic egg chamber mostly not present (40%)
Morphology								

LOC659226 L Primers: left: CCACAAAGTCGCCAAACATA right: CATTTCTCGTCTTCGGCTTT

	Phenotype after larval injection				Phenotype after pupal injection			
	Egg	Larvae	Pupae	Adult	Egg	Larvae	Pupae	Adult
Mortality 11 days	-	20%	20%		-	-	0%	0%
Mortality 22 days	-	30%	30%	30%	-	-		
Fertility				30%				
Morphology			Eclosion delayed	No living offspring	No cuticle (50-80% Not fertilized (30%)		Later than schedule	
